# Dumpster Diving in the Gut: Bacterial Microcompartments as Part of a Host-Associated Lifestyle

**DOI:** 10.1371/journal.ppat.1005558

**Published:** 2016-05-12

**Authors:** Christopher M. Jakobson, Danielle Tullman-Ercek

**Affiliations:** Department of Chemical and Biomolecular Engineering, University of California, Berkeley, Berkeley, California, United States of America; University of North Carolina at Chapel Hill School of Medicine, UNITED STATES

Upon arriving in the host gut, enteric pathogens face a daunting challenge: to proliferate in an environment already rich in commensal microbes and poor in available nutrients. Recent evidence suggests that *Salmonella* and other enteric bacteria conduct a coordinated assault employing two complimentary systems: bacterial microcompartments and the type III secretion system. While a portion of invading bacteria construct subcellular metabolic organelles designed to utilize unique nutrients, the remaining invading cells induce intestinal inflammation, remodeling the chemical environment of the gut to render it more favorable to *Salmonella* proliferation (Fig 1).

**Fig 1:**
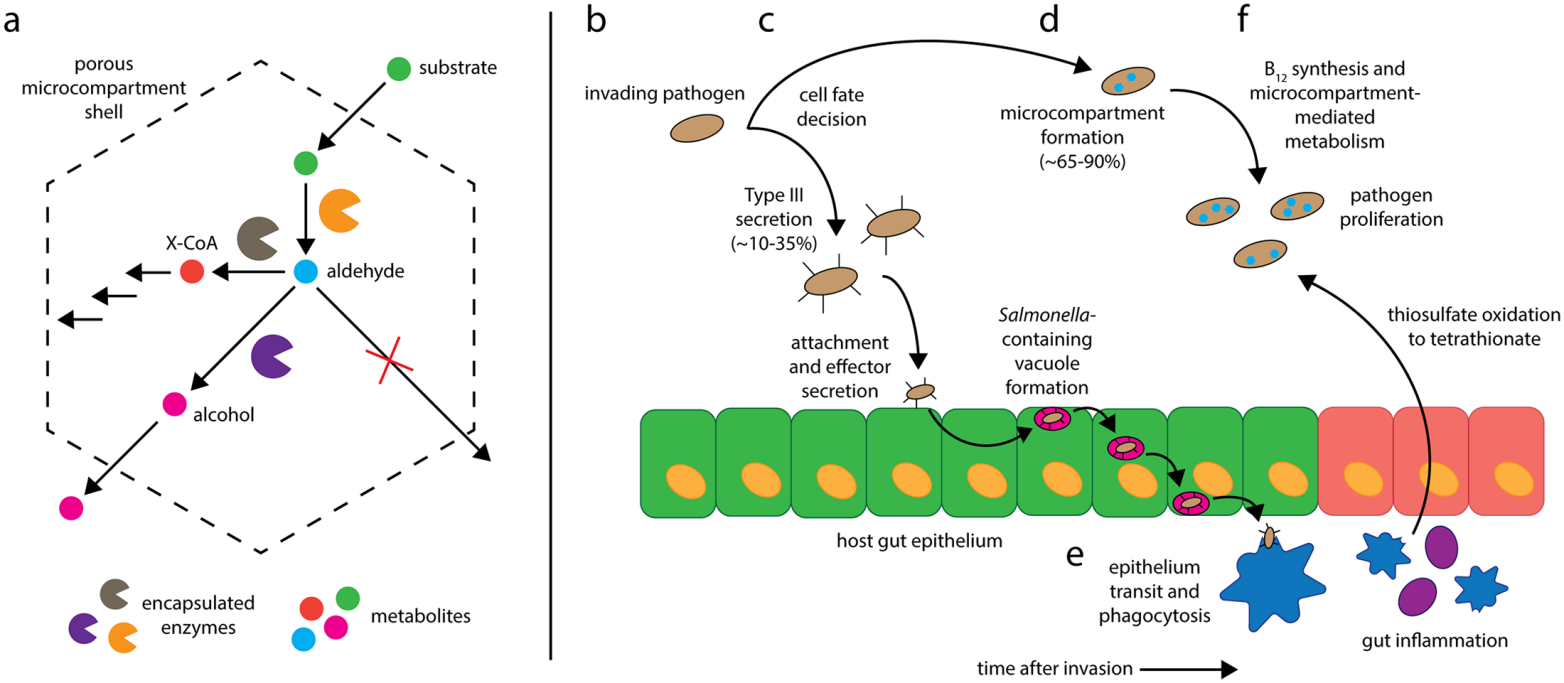
Bacterial microcompartment function and the coordinated invasion of the host gut by *Salmonella enterica*. (a): A substrate molecule enters the microcompartment and is converted to an aldehyde species, which is trapped in the microcompartment shell before being converted either to an alcohol or to a Coenzyme A-conjugated species [32]. (b) The invading pathogen population enters the gut. (c) Each pathogen cell undergoes a fate decision between type III secretion (~10%–35% of cells) and microcompartment formation (~65%–90% of cells). (d) Type III secretion-competent cells invade the host epithelium while microcompartment-competent cells form microcompartments and synthesize vitamin B_12_. (e) Type III secretion-competent cells traverse the epithelium and undergo phagocytosis in the lamina propria. (f) Gut inflammation causes thiosulfate oxidation to tetrathionate, allowing microcompartment-mediated metabolism and pathogen proliferation.

## What Are Bacterial Microcompartments and What Are They For?

Despite the received wisdom that eukarya possess intracellular organelles and bacteria do not, bacteria do use organelles called bacterial microcompartments to spatially segregate metabolism. Rather than a phospholipid membrane, however, these organelles are bound by a porous protein monolayer made up of trimeric, pentameric, and hexameric shell proteins. A suite of metabolic enzymes, including those required for cofactor regeneration, are encapsulated in the microcompartment lumen. The mechanism of microcompartment assembly remains elusive, but it is known that some enzymes are localized to the microcompartment through interactions with the inner face of the microcompartment shell [1,2]. Many pathogens possess microcompartments, including *Salmonella enterica*, *Escherichia coli*, *Listeria monocytogenes*, *Yersinia enterocolitica*, and *Shigella flexneri*, and microcompartment genes have been found in as many as 20% of sequenced bacterial genomes [3,4]. Bacteria are known to use a variety of microcompartment systems to metabolize compounds such as 1,2-propanediol [5], ethanolamine [6], and L-fucose and L-rhamnose [7]. All of these metabolic pathways proceed through toxic aldehyde intermediates, and it is proposed that the microcompartment shell functions to protect the rest of the bacterial cell contents from these toxic compounds as well as to sequester a private pool of the requisite cofactor molecules [8]. Compartmentalized metabolic processes may impart a competitive advantage to invading pathogens over the existing gut microbiota, which typically lack microcompartment operons and thus are unable to utilize the substrates metabolized in the microcompartments [9,10]. For example, *S*. *enterica* subsp. *enterica*, serovar Typhimurium dedicates approximately 2% of its genome to 1,2-propanediol and ethanolamine metabolism and the synthesis of associated cofactors, suggesting that these processes confer a significant competitive advantage at some point in the pathogen’s life cycle [11,12].

Microcompartment systems, such as the ethanolamine utilization microcompartment, enhance *E*. *coli* and *S*. *enterica* proliferation in diverse settings, including in food products, in a *Caenorhabditis elegans* model of infection, during growth on bovine intestinal content, and in the gut of a mouse model of *Salmonella* infection [9,13,14]. This indicates that there are many circumstances in which microcompartments may provide a competitive advantage to pathogens. Evidence suggests that the gut microbiota plays a central role in preventing host colonization by pathogens, possibly by sequestering critical nutrients [15]. In a model of *S*. Typhimurium infection of the mouse gut, for example, antibiotic treatment to reduce the abundance of native gut microbes renders the host more susceptible to *Salmonella* infection [16], and mice with a compromised microbiota clear *S*. Typhymurium from the gut much less effectively following nonfatal infection [17]. Gaining a unique metabolic capacity may help pathogens sidestep microbiotic defense mechanisms by creating a new nutritional niche in the host gut, but microcompartment-mediated metabolism also requires a unique micronutrient: the cofactor vitamin B_12_.

## The B_12_ Synthesis Paradox: Is 1,2-Propanediol Utilization an Aerobic or Anaerobic Process?

1,2-propanediol and ethanolamine utilization both require vitamin B_12_, and the B_12_ biosynthetic genes in *S*. *enterica* were found to be transcriptionally co-regulated with the 1,2-propanediol utilization operon [18]. This raised an apparent paradox: 1,2-propanediol and ethanolamine metabolism were once thought to occur only in aerobic conditions, whereas B_12_ synthesis is a strictly anaerobic process [11,19]. This puzzle was solved by the discovery that 1,2-propanediol and ethanolamine metabolism can proceed using tetrathionate as an electron acceptor in place of molecular oxygen [12]. Tetrathionate, in turn, is a product of the oxidation of thiosulfate, an abundant molecule in the gut produced by the inactivation of H_2_S. *Salmonella* or other pathogenic bacteria in the gut might thus synthesize vitamin B_12_ anaerobically while simultaneously respiring to tetrathionate instead of oxygen. Under normal gut conditions, oxidation of thiosulfate to tetrathionate is minimal; however, this reaction is accelerated by inflammation when the gut is rendered a more oxidizing environment [20]. The oxidizing environment of the inflamed gut may favor microcompartment-mediated metabolism by *Salmonella* and other pathogens, allowing their proliferation at the expense of the gut microbiota [14]. Indeed, many pathogenic bacteria possess a mechanism to induce just such an oxidative dysbiosis—the type III secretion system [21].

## Type III Secretion and Microcompartment-Mediated Metabolism: Does *Salmonella* Conduct a Coordinated Assault on the Gut Microbiota?

Upon encountering environmental cues that are indicative of the host intestinal tract (e.g., high osmolarity, low pH, and low oxygen concentration), many enteric pathogens, including *Salmonella* and *Shigella* spp., express type III secretion systems, which function to mechanically penetrate the intestinal epithelium and translocate various effector proteins into host cells [22]. These effectors not only mediate internalization of the *Salmonella* cell into a *Salmonella*-containing vacuole but also induce an inflammatory response throughout the host gut. This inflammatory response increases the rate of thiosulfate oxidation and hence the concentration of tetrathionate in the gut [23,20]. Not all the invading *Salmonella* cells, however, express the type III secretion system. Experiments examining the transcriptional regulation of type III secretion system promoters indicate that only a fraction of a given population expresses the type III secretion system even in appropriate inducing conditions [24]. What, then, is the role of the non-induced cells? This population is believed to remain in the gut in order to exploit the ensuing inflammation and gain a foothold in the metabolic competition between invaders and commensals [25,26].

Interestingly, 1,2-propanediol represses expression of the type III secretion system master regulator *hilA*, suggesting that cells may undergo a fate decision between type III secretion system-mediated epithelium invasion (leading to bacterial cell death) and 1,2-propanediol or ethanolamine metabolism (leading to proliferation) [23,27]. Furthermore, propionate, a downstream product of 1,2-propanediol metabolism, down-regulates another type III secretion system master regulator, HilD, at the post-translational level; it is proposed that endogenous propionate in the gut is primarily responsible for this phenomenon [28]. We additionally propose that post-translational modification of HilD as a result of intracellular propionate production may be a means of down-regulating type III secretion in response to 1,2-propanediol utilization microcompartment expression.

## Does the Paradigm of Nutritional Competition Extend beyond Micronutrients?

Nutritional immunity, the modulation of micronutrient concentrations by the host to prevent colonization by pathogens, is well characterized for transition metal micronutrients [29,30]. It seems likely that this paradigm, in which host and commensal processes are under selective pressure to sequester critical nutrients from pathogens, extends beyond micronutrients such as iron and copper ions to other small molecules as well. Bacterial microcompartments may therefore represent another step in the nutritional arms race between pathogens and commensal species. The coordinated induction of the type III secretion system and bacterial microcompartments in separate bacterial populations allows the pathogen population to induce and exploit an inflamed state in the host gut, allowing colonization followed by diarrhea favorable for subsequent transmission to other hosts [31]. The proposed interplay between type III secretion and bacterial microcompartments suggests that pathogens “dumpster diving” in the gut can develop specialized metabolic mechanisms to utilize compounds otherwise considered to be waste by the gut microbiota. These strategies may involve the coordinated action of multiple cellular processes across the invading bacterial population.
